# Supplementary material for: Single-cell bisulfite-free 5mC and 5hmC sequencing with high sensitivity and scalability
Source: Proc Natl Acad Sci U S A. 2023 Nov 27;120(49):e2310367120. doi: 10.1073/pnas.2310367120 (PMC10710054; doi:10.1073/pnas.2310367120)
Supplement: Supplementary file 1 — Appendix 01 (PDF) [file pnas.2310367120.sapp.pdf]

# Supporting Information for

## Single-cell bisulfite-free 5mC and 5hmC sequencing with high sensitivity and scalability

Yunlong Cao[a,b,1], Yali Bai[b,c,1], Tianjiao Yuan[a,1], Liyang Song[b], Yu Fan[b], Liuhao Ren[a], Weiliang Song[a], Jiahui Peng[a], Ran An[b], Qingqing Gu[b], Yinghui Zheng[a], Xiaoliang Sunney Xie[a,b,d,*]

[a]School of Life Sciences, Biomedical Pioneering Innovation Center (BIOPIC), Peking University, Beijing 100871, P.R. China.

[b]Changping Laboratory, Beijing, P.R. China.

[c]Joint Graduate Program of Peking-Tsinghua-NIBS, School of Life Sciences, Tsinghua University, Beijing 100084, China.

[d]Beijing Advanced Innovation Center for Genomics, Peking University, Beijing, P.R. China.

[1]These authors contribute equally to this work.

*Xiaoliang Sunney Xie

**Email:** sunneyxie@biopic.pku.edu.cn

**This PDF file includes:**

Figures S1 to S16

Tables S1 to S5

**Other supporting materials for this manuscript include the following:**

Datasets S1 to S6

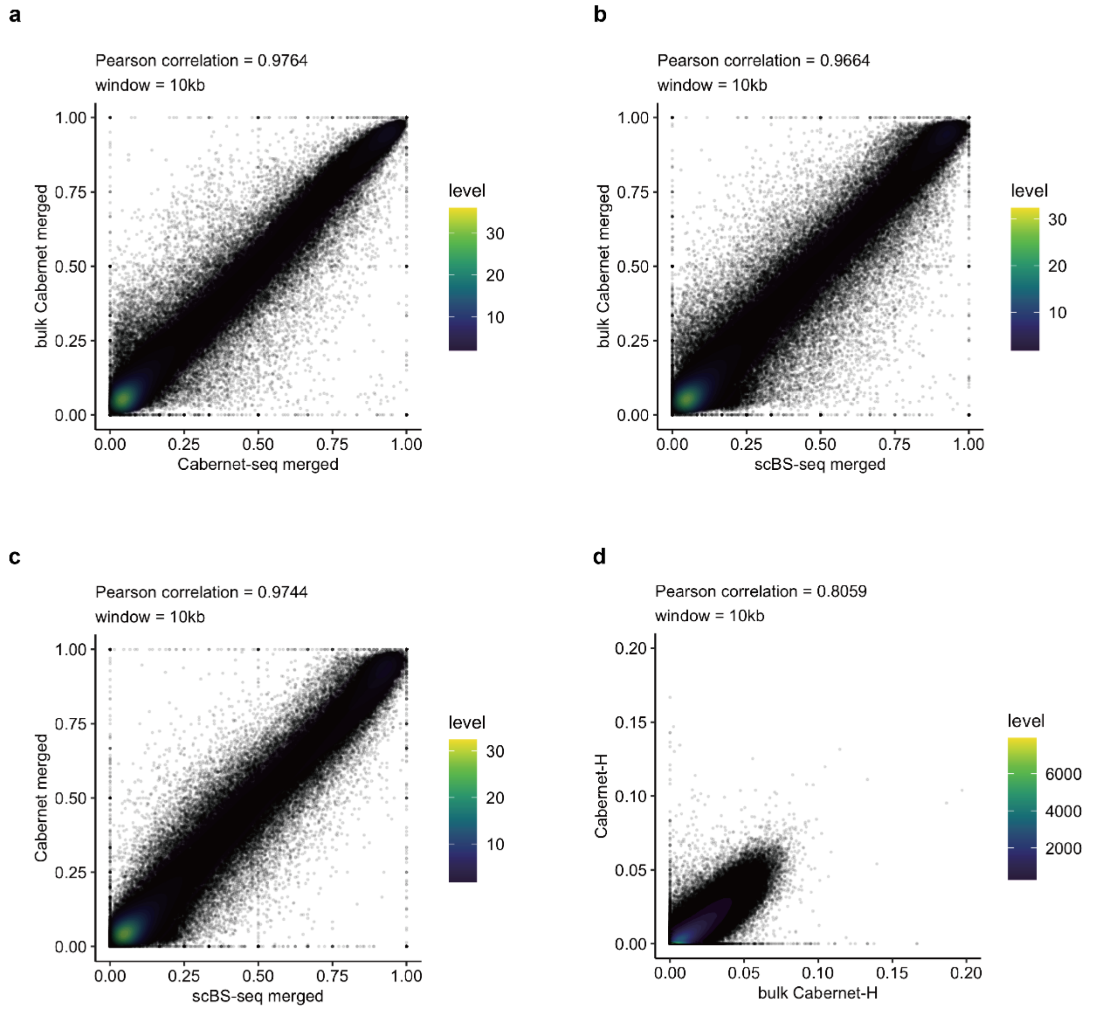


**Fig. S1. Correlation between different methylome sequencing methods. (a)** Correlation density plot of signals between Cabernet (merged from single cells) and Cabernet_bulk (bulk) in K562 cells. Correlation analysis was performed with 10-kb bins spanning the genome. **(b)** Correlation density plot between Cabernet_bulk signals and scBS-seq signals in K562 cells (in 10-kb bins). **(c)** Correlation density plot between Cabernet signals and scBS-seq signals in K562 cells (in 10-kb bins). **(d)** Correlation density plot of Cabernet-H signals (single cell) and Cabernet-H_bulk (bulk) in mESCs (in 10-kb bins).

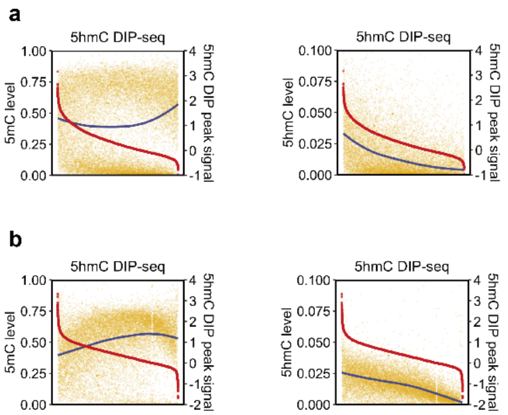


**Fig. S2. (a)** Left: signal intensities of 5hmC DIP-Seq peaks (red) within promoter regions in mESCs and 5mC levels detected by Cabernet (blue) at the corresponding peak regions. Right: signal intensities of 5hmC DIP-Seq peaks (red) within promoter regions in mESCs and 5hmC levels (blue) at the corresponding peak regions. **(b)** Left: signal intensities of 5hmC DIP-Seq peaks (red) within gene body regions in mESCs and 5mC levels detected by Cabernet (blue) at the corresponding peak regions. Right: signal intensities of 5hmC DIP-Seq peaks (red) within gene body regions in mESCs and 5hmC levels (blue) at the corresponding peak regions.

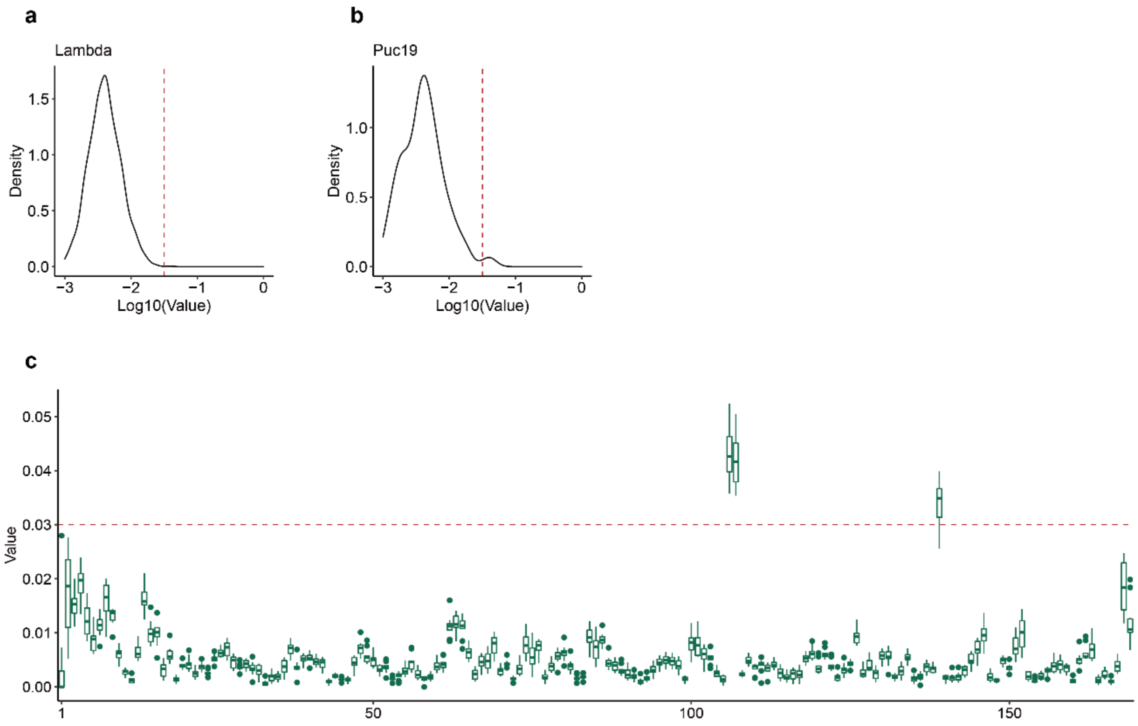


**Fig. S3. Overview of Cabernet-H data quality. (a)** Density distribution of 5hmC meanvalue detected by Cabernet-H in lambda DNA. The read dotted line is at log10(0.03). **(b)** Density distribution of 5hmC meanvalue detected by Cabernet-H in puc19. The read dotted line is at log10(0.03). **(c)** Meanvalue of 5hmC at each CpG sites detected by Cabernet-H in puc19.

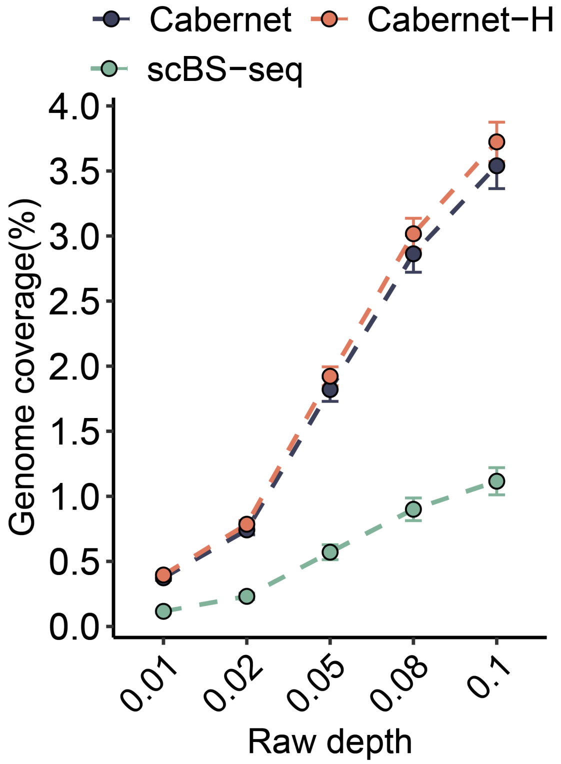


**Fig. S4.** Genome coverage of Cabernet, Cabernet-H and scBS-seq under different number of downsampled reads in K562 cells.

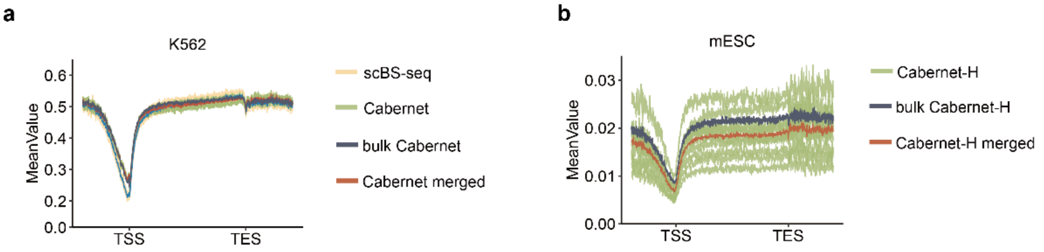


**Fig. S5. Modification pattern of 5mC and 5hmC detected by Cabernet and Cabernet-H on gene body. (a)** Modification pattern of 5mC detected by Cabernet and scBS-seq on gene body in K562 cells. Averaged DNA methylation levels along gene bodies between 2 kilobase (kb) upstream of the transcription start sites (TSS) and 2 kb downstream of the transcription end sites (TES) of all RefSeq genes. Green: Cabernet; red: Cabernet_merged; gray: Cabernet_bulk; yellow: scBS-seq. **(b)** Modification pattern of 5hmC detected by Cabernet-H on gene body in mESCs. Averaged 5hmC levels along the gene bodies between 2 kb upstream of the transcription start sites (TSS) and 2 kb downstream of the transcription end sites (TES) of all RefSeq genes. Green: Cabernet-H; red: Cabernet-H_merged; gray: Cabernet-H_bulk.

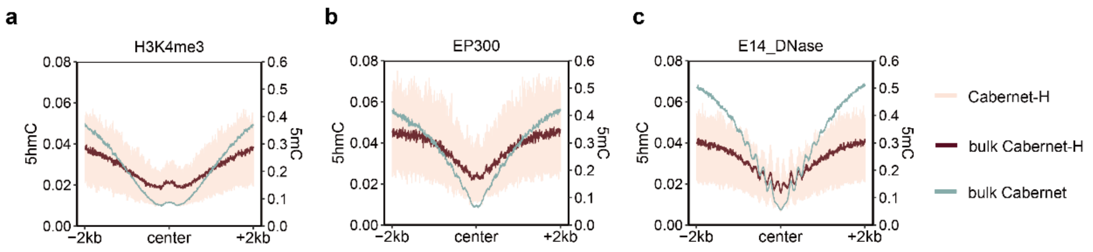


**Fig. S6. Enrichment results of Cabernet and Cabernet-H at binding regions of different factors. (a)** Enrichment characteristics of Cabernet and Cabernet-H at H3K4me3 binding region (H3K4me3 peak center ±2kb). Pink: Cabernet-H; brown: Cabernet-H_bulk; green: Cabernet_bulk. **(b)** Enrichment characteristics of Cabernet and Cabernet-H at EP300 binding region (EP300 peak center ±2kb). Pink: Cabernet-H; brown: Cabernet-H_bulk; green: Cabernet_bulk. **(c)** Enrichment characteristics of Cabernet and Cabernet-H at E14_DNase binding region (DNase peak center ±2kb).

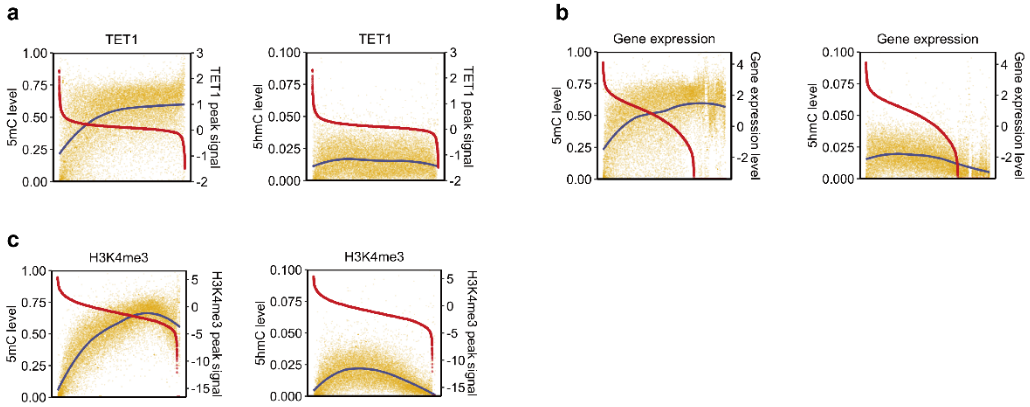


**Fig. S7. Comparison between 5mC/5hmC levels detected by Cabernet/Cabernet-H and signal intensities of different factors at gene body region. (a)** Left: signal intensities of Tet1 ChIP-Seq peaks (red) within gene body regions in mESCs and the 5mC levels detected by Cabernet (blue) at the corresponding peak regions. The horizontal axis from left to right of each box represents the Tet1 peaks, which overlapped with gene body regions, ranked by peak signal intensities from high to low. Right: signal intensities of Tet1 ChIP-Seq peaks within gene body regions in mESCs and the DNA 5hmC levels at the corresponding peak regions. **(b)** Left: 5mC levels (blue) at gene body regions and the expression levels of corresponding genes (red) in mESCs. The log10 of gene expression levels (transcripts per kilobase per million mapped reads, TPM) were calculated and presented. Right: DNA 5hmC levels at gene body regions and the expression levels of corresponding genes in mESCs. **(c)** Left: signal intensities of H3K4me3 ChIP-Seq peaks (red) within gene body regions in mESCs and 5mC levels (blue) at the corresponding peak regions. Right: signal intensities of H3K4me3 ChIP-Seq peaks (red) within gene body regions in mESCs and 5hmC levels (blue) at the corresponding peak regions.

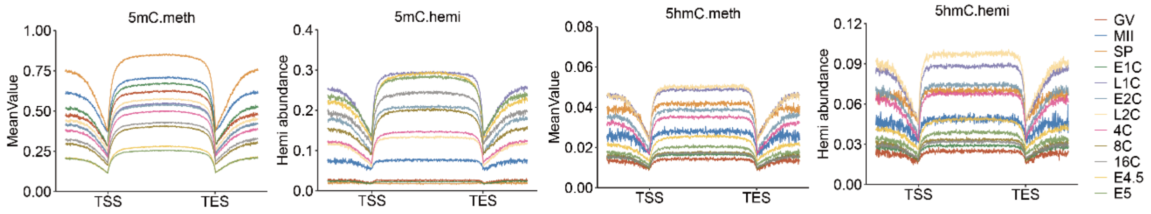


**Fig. S8. Key features of DNA methylome in early mouse embryos.** Distribution of 5mC/hemi-5mC, 5hmC/hemi-5hmC detected by Cabernet/Cabernet-H in early mouse embryos on gene body regions between 2 kilobase (kb) upstream of the transcription start sites (TSS) and 2-kb downstream of the transcription end sites (TES) of all RefSeq genes. Different colors represent different stages during embryonic development.

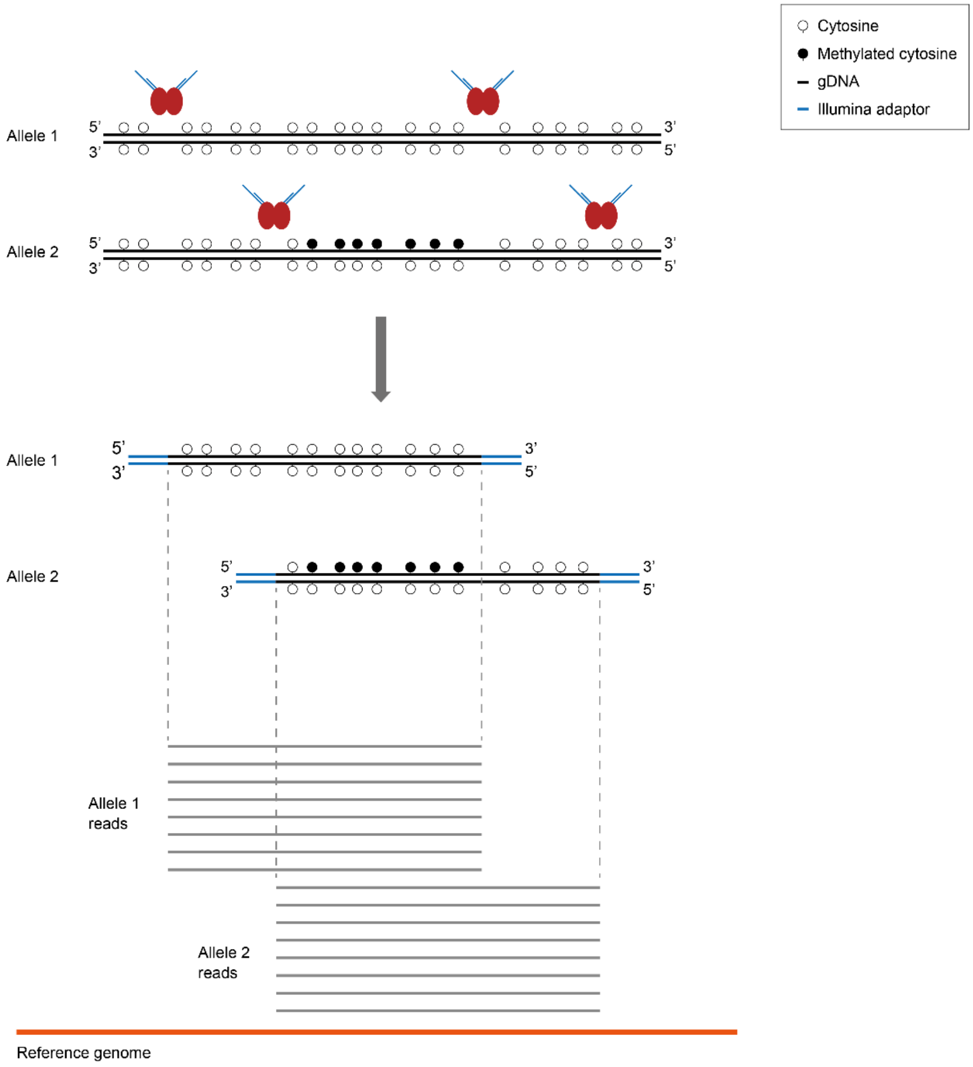


**Fig. S9. Principle for allele counting in Cabernet.** The amplicons aligned to the same starting and ending sites on the reference genome are originated from the same allele of the single-cell genomic DNA. This allows for the detection of hemi-methylation in each allele.

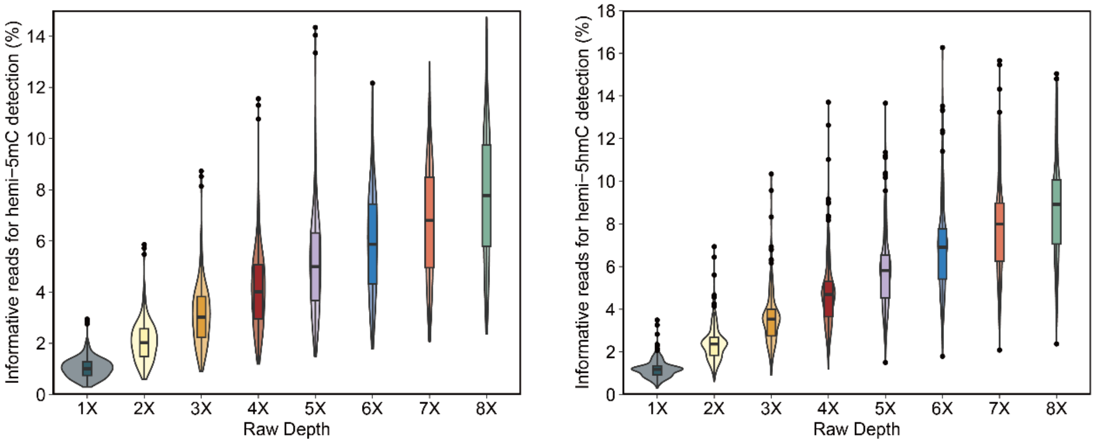


**Fig. S10. Average fraction of reads from each cell that are informative of hemi-5mC and hemi-5hmC at varied sequencing depths.**

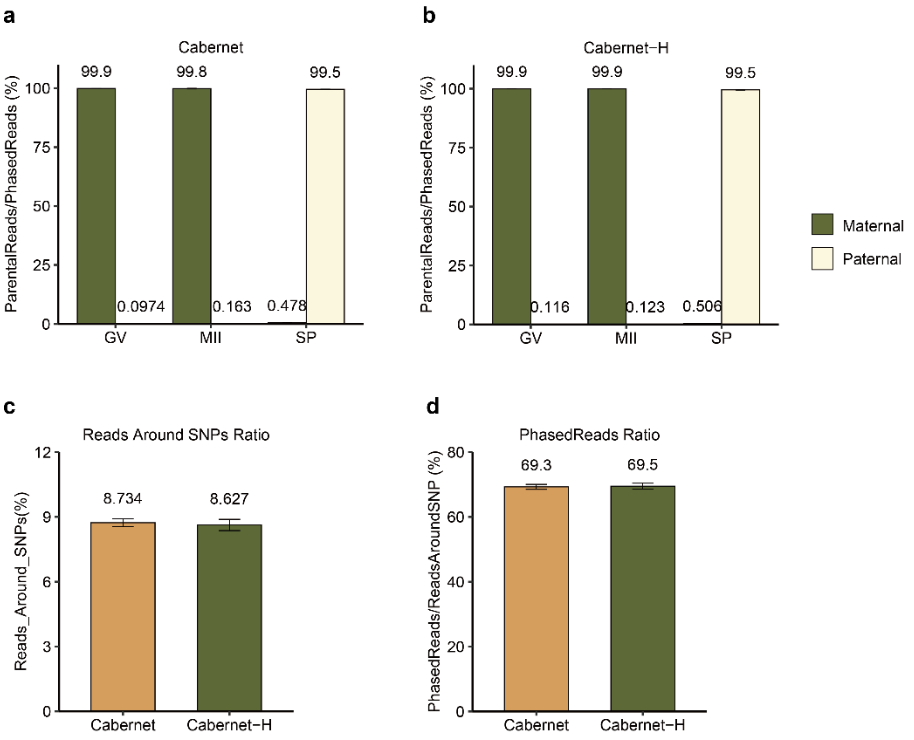


**Fig. S11. Accuracy of parental phasing for Cabernet and Cabernet-H. (a)** The ratio of maternal/ paternal reads to all phased reads in GV/MII/SP cells sequenced by Cabernet. **(b)** The ratio of maternal/ paternal reads to all phased reads in GV/MII/SP cells sequenced by Cabernet-H. **(c)** The percentage of reads around SNPs in all reads. **(d)** The ratio of phased reads to all reads around SNPs.

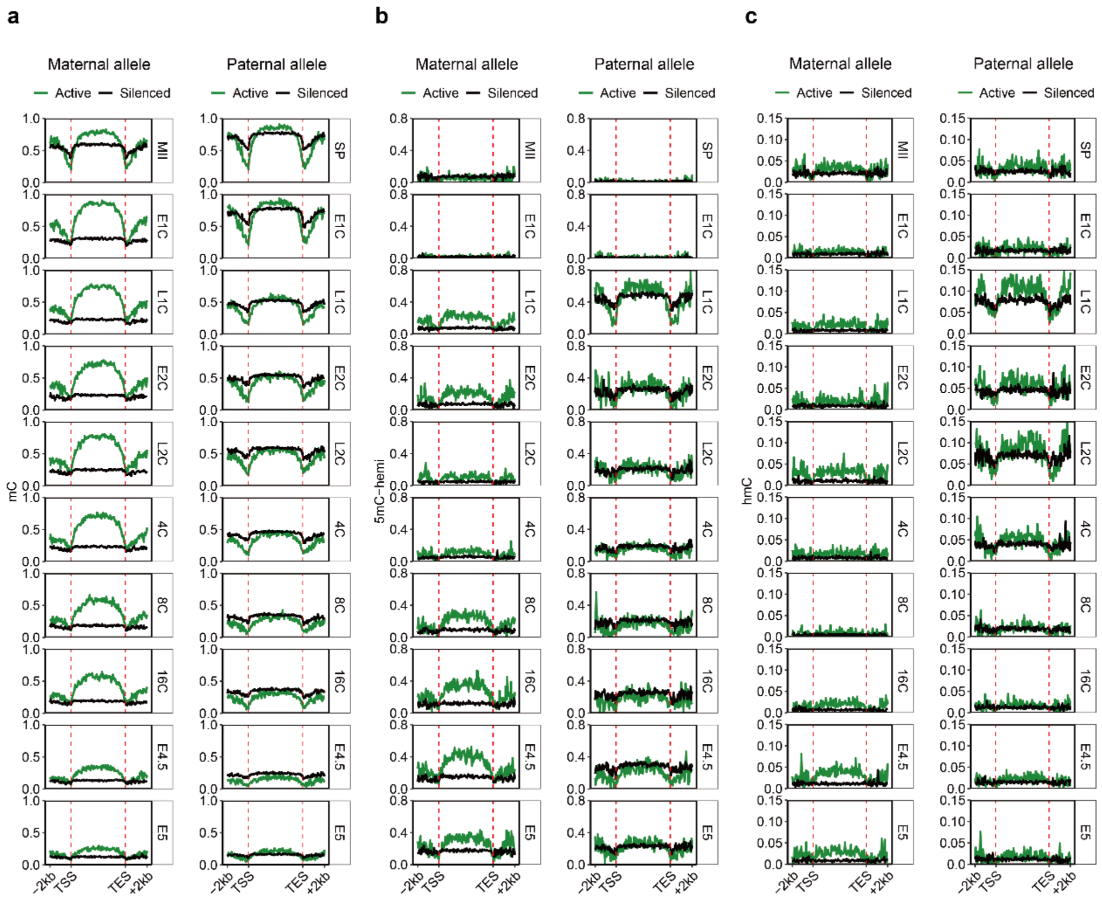


**Fig. S12. Methylation levels at gene body of active/silenced genes in the maternal/paternal genome of oocytes/sperm cells and early mouse embryos. (a)** 5mC level at gene body of active/silenced genes in the maternal (left) and paternal genome (right). **(b)** hemi-5mC abundance at gene body of active/silenced genes in the maternal (left) and paternal genome (right). **(c)** 5hmC level at gene body of active/silenced genes in the maternal (left) and paternal genome (right).

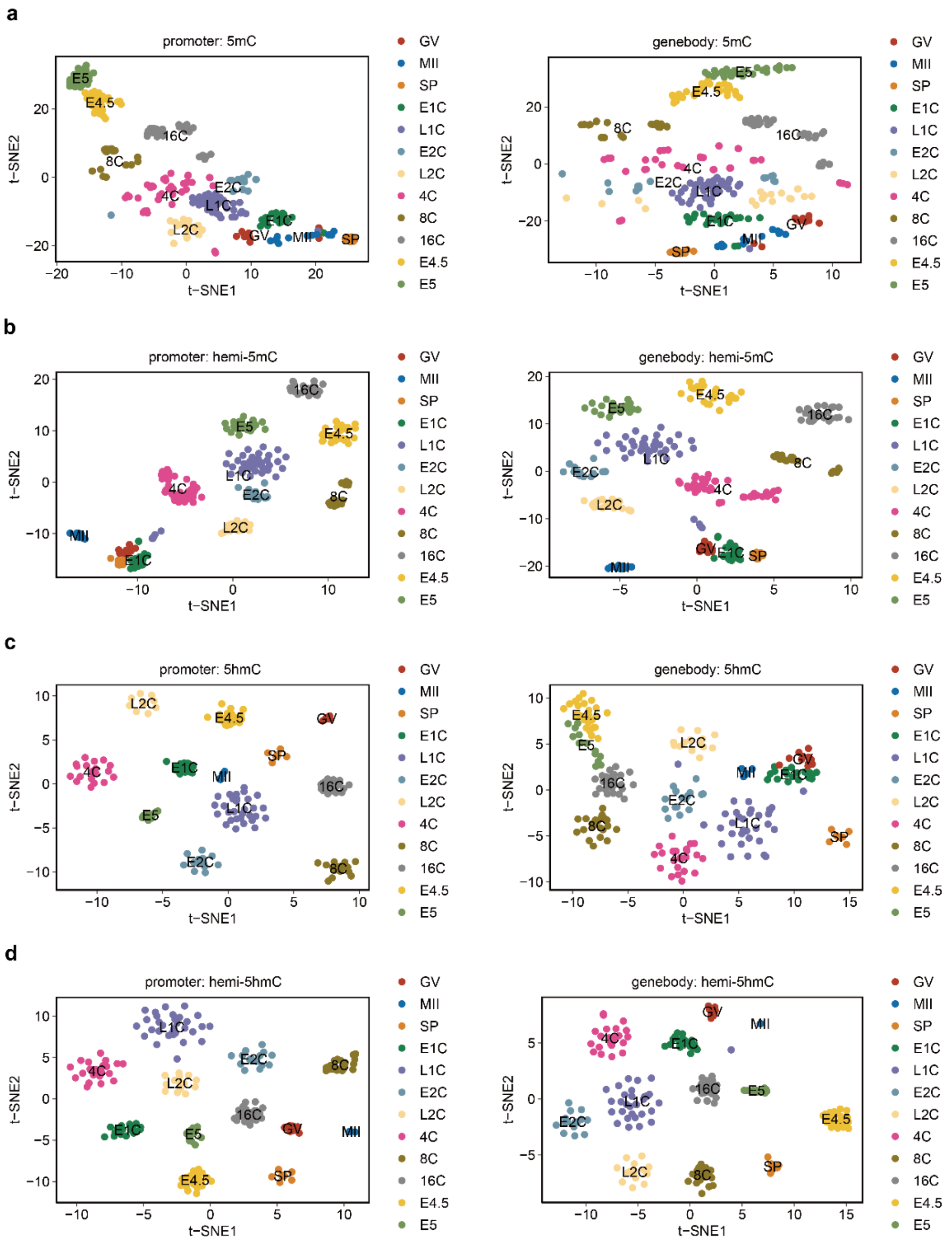


**Fig. S13. t-SNE analysis of cells based on 5mC/5hmC/hemi-5mC/hemi-5hmC. Colors indicate different cell stages. (a)** t-SNE plot of 5mC level at promoter (left) and gene body (right). **(b)** t-SNE plot of hemi-5mC abundance at promoter (left) and gene body (right). **(c)** t-SNE plot of 5hmC level at promoter (left) and gene body (right). **(d)** t-SNE plot of hemi-5hmC abundance at promoter (left) and gene body (right).

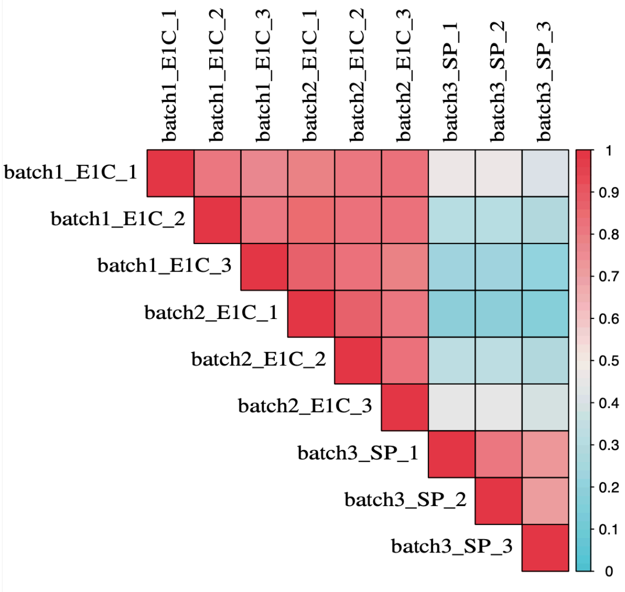


**Fig. S14. Heatmap of Pearson correlation of 5mC levels between two different batches of early 1-cells and sperm cells.**

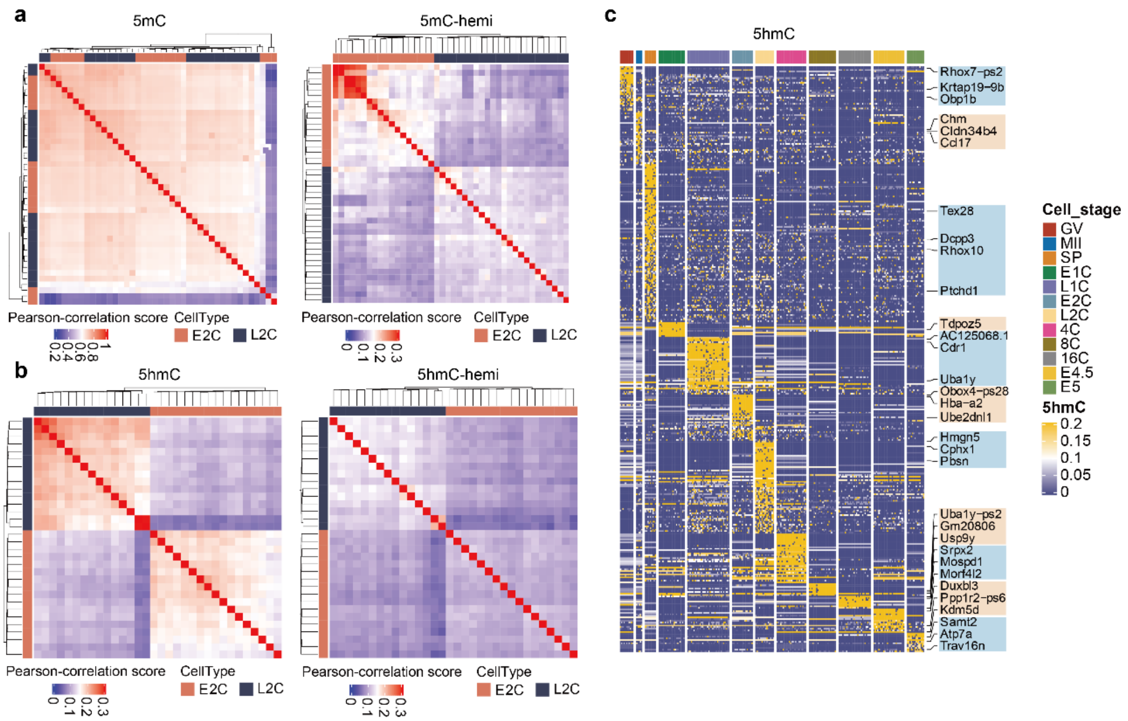


**Fig. S15. Key features of DNA methylome during early mouse embryos. (a)** Left: clustered heatmap showing Pearson correlation between 5mC abundance of different cells at early-2-cell stage (E2C) and late-2-cell stage (L2C). Right: clustered heatmap showing Pearson correlation between hemi-5mC abundance of different cells at E2C stage and L2C stage. **(b)** Left: clustered heatmap showing Pearson correlation between 5hmC abundance of different cells at E2C stage and L2C stage. Right: clustered heatmap showing Pearson correlation between hemi-5hmC abundance of different cells at E2C stage and L2C stage. **(c)** Abundance of 5hmC modification on gene body regions at different developmental stages.

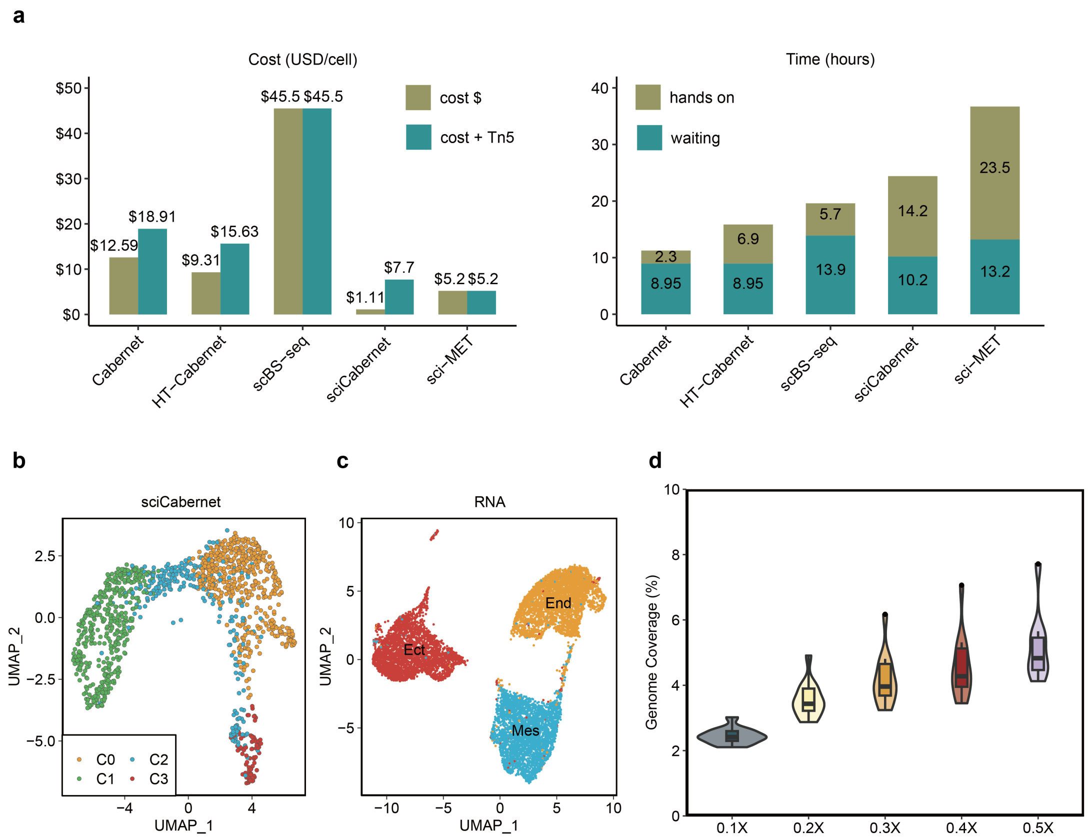


**Fig. S16. Cost-effectiveness and performance of Cabernet technology. (a)** Cost of money and time by different sequencing methods. The yellow bar refers to the cost of reagents; the green bar refers to the combined cost of reagents plus Tn5. Detailed calculations of cost are shown in Dataset S6. **(b)** UMAP showing the clustering of E7.5 mouse embryo cells sequenced by sci-Cabernet. **(c)** UMAP showing the clustering of E7.5 mouse embryo cells based on 10x scRNA-seq data. **(d)** Violin plot showing the genome coverage of sci-Cabernet at different sequencing depths in K562 cells.

**Table S1.** Cabernet Transposase-loaded Oligos (5'-3')

| | |
|---|---|
| **Reverse complement:** | (5phos)CTGTCTCTTATACACATCT |
| **Me-P5 adaptor** | T/5Me-dC/GT/5Me-dC/GG/5Me-dC/AG/5Me-dC/GT/5Me-dC/AGATGTGTATAAGAGACAG |
| **Me-P7 adaptor** | GT/5Me-dC/T/5Me-dC/GTGGG/5Me-dC/T/5Me-dC/GGAGATGTGTATAAGAGACAG |
| **HydMe-P5 adaptor** | T/5HydMe-dC/GT/5HydMe-dC/GG/5HydMe-dC/AG/5HydMe-dC/GT/5HydMe-dC/AGATGTGTATAAGAGACAG |
| **HydMe-P7 adaptor** | GT/5HydMe-dC/T/5HydMe-dC/GTGGG/5HydMe-dC/T/5HydMe-dC/GGAGATGTGTATAAGAGACAG |

**Table S2.** Cabernet Index PCR Primers (5'-3')

| | |
|---|---|
| **Index primer N701** | CAAGCAGAAGACGGCATACGAGATTCGCCTTAGTCTCGTGGGCTCGG |
| **Index primer N702** | CAAGCAGAAGACGGCATACGAGATCTAGTACGGTCTCGTGGGCTCGG |
| **Index primer N703** | CAAGCAGAAGACGGCATACGAGATTTCTGCCTGTCTCGTGGGCTCGG |
| **Index primer N704** | CAAGCAGAAGACGGCATACGAGATGCTCAGGAGTCTCGTGGGCTCGG |
| **Index primer N705** | CAAGCAGAAGACGGCATACGAGATAGGAGTCCGTCTCGTGGGCTCGG |
| **Index primer N706** | CAAGCAGAAGACGGCATACGAGATCATGCCTAGTCTCGTGGGCTCGG |
| **Index primer N707** | CAAGCAGAAGACGGCATACGAGATGTAGAGAGGTCTCGTGGGCTCGG |
| **Index primer N708** | CAAGCAGAAGACGGCATACGAGATCCTCTCTGGTCTCGTGGGCTCGG |
| **Index primer N709** | CAAGCAGAAGACGGCATACGAGATAGCGTAGCGTCTCGTGGGCTCGG |
| **Index primer N710** | CAAGCAGAAGACGGCATACGAGATCAGCCTCGGTCTCGTGGGCTCGG |
| **Index primer N711** | CAAGCAGAAGACGGCATACGAGATTGCCTCTTGTCTCGTGGGCTCGG |
| **Index primer N712** | CAAGCAGAAGACGGCATACGAGATTCCTCTACGTCTCGTGGGCTCGG |
| **Index primer N501** | AATGATACGGCGACCACCGAGATCTACACTAGATCGCTCGTCGGCAGCGTC |

| | |
|---|---|
| **Index primer N502** | AATGATACGGCGACCACCGAGATCTACACCTCTCTATTCGTCGGCAGCGTC |
| **Index primer N503** | AATGATACGGCGACCACCGAGATCTACACTATCCTCTTCGTCGGCAGCGTC |
| **Index primer N504** | AATGATACGGCGACCACCGAGATCTACACAGAGTAGATCGTCGGCAGCGTC |
| **Index primer N505** | AATGATACGGCGACCACCGAGATCTACACGTAAGGAGTCGTCGGCAGCGTC |
| **Index primer N506** | AATGATACGGCGACCACCGAGATCTACACACTGCATATCGTCGGCAGCGTC |
| **Index primer N507** | AATGATACGGCGACCACCGAGATCTACACAAGGAGTATCGTCGGCAGCGTC |
| **Index primer N508** | AATGATACGGCGACCACCGAGATCTACACCTAAGCCTTCGTCGGCAGCGTC |

**Table S3.** sciCabernet Transposase-loaded Oligos (5Me-dC, 5'-3')

| **Reverse Complement:** | (5phos)CTGTCTCTTATACACATCT | | |
|---|---|---|---|
| **Name** | **i5_PCR** | **i5_barcode** | **Mosaic end** |
| **sciCabernet_P5_Tn5_1** | TCGTCGGCAGCGTCTGGGTT | TGCG | AGATGTGTATAAGAGACAG |
| **sciCabernet_P5_Tn5_2** | TCGTCGGCAGCGTCTGGGTT | ATACG | AGATGTGTATAAGAGACAG |
| **sciCabernet_P5_Tn5_3** | TCGTCGGCAGCGTCTGGGTT | GATATGAT | AGATGTGTATAAGAGACAG |
| **sciCabernet_P5_Tn5_4** | TCGTCGGCAGCGTCTGGGTT | CCGTCTATC | AGATGTGTATAAGAGACAG |
| **sciCabernet_P5_Tn5_5** | TCGTCGGCAGCGTCTGGGTT | CCGC | AGATGTGTATAAGAGACAG |
| **sciCabernet_P5_Tn5_6** | TCGTCGGCAGCGTCTGGGTT | TGTAG | AGATGTGTATAAGAGACAG |
| **sciCabernet_P5_Tn5_7** | TCGTCGGCAGCGTCTGGGTT | ATGTAGTC | AGATGTGTATAAGAGACAG |
| **sciCabernet_P5_Tn5_8** | TCGTCGGCAGCGTCTGGGTT | GAACTCGGC | AGATGTGTATAAGAGACAG |
| **sciCabernet_P5_Tn5_9** | TCGTCGGCAGCGTCTGGGTT | GAGT | AGATGTGTATAAGAGACAG |
| **sciCabernet_P5_Tn5_10** | TCGTCGGCAGCGTCTGGGTT | GGTA | AGATGTGTATAAGAGACAG |
| **sciCabernet_P5_Tn5_11** | TCGTCGGCAGCGTCTGGGTT | TAGTAA | AGATGTGTATAAGAGACAG |

| | | | |
|---|---|---|---|
| **sciCabernet_P5_Tn5_12** | TCGTCGGCAGCGTCTGGGTT | GTAG | AGATGTGTATAAGAGACAG |
| **Name** | **i7_PCR** | **i7_barcode** | **Mosaic end** |
| **sciCabernet_P7_Tn5_1** | GTCTCGTGGGCTCGGCTGTCC | CCTAG | AGATGTGTATAAGAGACAG |
| **sciCabernet_P7_Tn5_2** | GTCTCGTGGGCTCGGCTGTCC | AGAGC | AGATGTGTATAAGAGACAG |
| **sciCabernet_P7_Tn5_3** | GTCTCGTGGGCTCGGCTGTCC | CTACTATAC | AGATGTGTATAAGAGACAG |
| **sciCabernet_P7_Tn5_4** | GTCTCGTGGGCTCGGCTGTCC | TATC | AGATGTGTATAAGAGACAG |
| **sciCabernet_P7_Tn5_5** | GTCTCGTGGGCTCGGCTGTCC | TTGATGGCC | AGATGTGTATAAGAGACAG |
| **sciCabernet_P7_Tn5_6** | GTCTCGTGGGCTCGGCTGTCC | AGTT | AGATGTGTATAAGAGACAG |
| **sciCabernet_P7_Tn5_7** | GTCTCGTGGGCTCGGCTGTCC | GTTCGAAAG | AGATGTGTATAAGAGACAG |
| **sciCabernet_P7_Tn5_8** | GTCTCGTGGGCTCGGCTGTCC | TTAT | AGATGTGTATAAGAGACAG |

**Table S4.** sciCabernet Pre-amplification Primers (5'-3')

| | |
|---|---|
| **Pre-amp forward primer** | TCGTCGGCAGCGTCTGGGTT |
| **Pre-amp reverse primer** | GTCTCGTGGGCTCGGCTGTCC |

**Table S5.** sciCabernet Sequencing Primers (LNA, 5'-3')

| | |
|---|---|
| **sciCabernet_Read1** | TCGTCGGCAGCGTCTGGGTT |
| **sciCabernet_Read2** | GTCTCGTGGGCTCGGCTGTCC |
| **sciCabernet_index1** | GGACAGCCGAGCCCACGAGAC |
| **sciCabernet_index2** | AACCCAGACGCTGCCGACGA |

**Dataset S1 (separate file).** Metadata of Cabernet, Cabernet-H and scWGBS on K562 cell line

**Dataset S2 (separate file).** Metadata of Cabernet-H on mESC

**Dataset S3 (separate file).** Metadata of Cabernet/Cabernet-H on different embryo stages

**Dataset S4 (separate file).** Metadata of sciCabernet squencing

**Dataset S5 (separate file).** Downloaded data information

**Dataset S6 (separate file).** Cost of money by different sequencing methods
